# Differentiation of Human Protein-Induced Pluripotent Stem Cells toward a Retinal Pigment Epithelial Cell Fate

**DOI:** 10.1371/journal.pone.0143272

**Published:** 2015-11-25

**Authors:** Jie Gong, Mark A. Fields, Ernesto F. Moreira, Hannah E. Bowrey, Monika Gooz, Zsolt Ablonczy, Lucian V. Del Priore

**Affiliations:** 1 Department of Ophthalmology, Storm Eye Institute, Medical University of South Carolina, Charleston, SC, United States of America; 2 Department of Regenerative Medicine and Cell Biology, Medical University of South Carolina, Charleston, SC, United States of America; 3 Department of Drug Discovery and Biomedical Sciences, Medical University of South Carolina, Charleston, SC, United States of America; University of Florida, UNITED STATES

## Abstract

Compared with many induced pluripotent stem cell (iPSC) lines generated using retrovirus and other non-integrating methods, the utilization of human protein-induced iPSC (piPSC) lines may provide a safer alternative for the generation of retinal pigment epithelial (RPE) cells for transplantation in retinal degenerative diseases. Here we assess the ability of piPSCs to differentiate into RPE cells, and to perform native RPE cell behavior. piPSCs were seeded in 6-well low-attachment plates to allow embryoid body formation, and then analyzed for pluripotent stem cell markers NANOG, SSEA4 and TRA-1-60 by immunofluorescence. Following colony formation, piPSCs were assessed for confirmation of RPE cell differentiation by staining for zonula occludens (ZO-1), bestrophin, microphthalmia-associated transcription factor (MITF) and retinal pigment epithelium specific protein-65 (RPE65). To evaluate piPSC-RPE cell phagocytic ability, adult bovine photoreceptor rod outer segments (ROS) were fed to piPSC-RPE cells, which were analyzed by fluorescent microscopy and flow cytometry. Undifferentiated piPSCs expressed all pluripotent markers assessed and formed embryoid body aggregates after 7 days. Differentiated piPSC-RPE cells expressed ZO-1, bestrophin, MITF and RPE65, typical RPE cell markers. Flow cytometry revealed robust ingestion of fluorescently-labeled ROS by piPSC-RPE cells, which was over four-times greater than that of undifferentiated piPSCs and comparable to that of an immortalized RPE cell line. Phagocytosis activity by piPSC-RPE cells was significantly reduced after the addition of anti-integrin αVβ5. In conclusion, piPSCs can be differentiated toward an RPE cell fate, expressing RPE cell markers and resembling native RPE cells in behavior. These results demonstrate that piPSCs can be differentiated into RPE-like cells using a method that has an increased safety profile, a critical consideration for the development of better treatments for retinal degenerative diseases such as age-related macular degeneration (AMD).

## Introduction

Age-related macular degeneration (AMD) is a leading cause of blindness in the United States and Western Europe, and it will become an increasing burden as the population ages [1, 2]. There are two forms of AMD. The exudative or “wet” type is characterized by neovascularization of the choroid and affects 10% of AMD patients [3]. Currently, this form of AMD can be controlled with intravitreal injections of vascular endothelial growth factor inhibitors. The dry type is more common, representing the majority of individuals with AMD [3]. In both types of AMD, the disease is characterized by dysfunction and eventual loss of retinal pigment epithelial (RPE) cells, a critical cell type in the maintenance of retinal function [4–9]. Despite advances in the treatment of the exudative type, presently there are no sight-restoring therapies available for patients with the dry type AMD. Recent studies demonstrate the safety of human embryonic stem cells (ESCs) transplanted into the subretinal space in patients with atrophy from advanced AMD and Stargardt disease [10, 11]. However, transplantation of allografts requires the use of immune suppression which is not well-tolerated in elderly individuals with atrophic AMD [12]. Novel methods for RPE cell generation using patient-specific strategies may avoid the need for immune suppression and thereby provide an advantage over ESCs.

Several methods for the development of RPE cell lines have been demonstrated. For example, RPE-like cells generated from human ESCs express RPE cell markers such as zonula occludens protein-1 (ZO-1), RPE-specific protein-65 (RPE65), cellular retinaldehyde-binding protein (CRALBP), and c-mer proto-oncogene tyrosine kinase (Mertk) [13, 14]. These cells behave in a manner similar to primary RPE cells, both in culture and in situ [15, 16]. Induced pluripotent stem cells (iPSCs) can be generated via the expression of OCT4, NANOG, Sox2 and Lin28 [17, 18], using lentiviral and retroviral methods. Generating iPSCs using these methods can cause multiple chromosomal integrations and possible genetic dysfunction [19–21], creating additional obstacles for clinical therapy. Therefore, it is important to establish novel approaches for generating iPSCs free from such limitations. Delivering factors as proteins eliminates the risks associated with retroviral integration by taking advantage of a DNA vector-free protein transduction system [21, 22]. This approach carries an increased safety profile when considering clinical trials [21, 23]. While human protein-induced iPSCs (piPSCs) have been used to generate cell types such as dopamine neurons [22], to our knowledge this approach has not previously been utilized for the generation of piPSC-derived RPE cells.

Here we demonstrate confirmatory evidence that piPSCs can be induced to differentiate toward an RPE cell fate, expressing typical RPE cell markers, and robust phagocytic ability. This is an important step in establishing an immune matched, functional RPE cell donor tissue, free from limitations of chromosomal integrations and immune rejection.

## Materials and Methods

### piPSC culture

Human piPSCs were purchased from System Biosciences (Catalogue number: SC801A-1, Mountain View, CA). The method for their original generation, which was since licensed by System Biosciences, and which used human newborn fibroblasts, is described in detail elsewhere [21].

Approximately 0.5 × 10^6^ human piPSCs were plated into two, 9.5 cm^2^ wells of a six-well culture plate (Corning Life Sciences, Acton, MA) containing a feeder layer of irradiated CF-1 mouse embryonic fibroblasts (MEF, American Type Culture Collection (ATCC), Manassas, VA). Cells were cultured at 37°C, 5% CO_2_, in an incubator with human iPSC medium containing knockout Dulbecco’s Modified Eagle’s Medium: Nutrient Mixture F12 (DMEM/F12, Invitrogen-Gibco, Life Technologies, Grand Island, NY) supplemented with 20% knockout serum replacement (KSR, Invitrogen-Gibco, Life Technologies), GLUTAMAX (2.0 mM, Invitrogen-Gibco, Life Technologies), minimum essential medium (MEM) non-essential amino acids (0.1 mM, Invitrogen-Gibco, Life Technologies), β-mercaptoethanol (β-ME, 0.1 mM, Sigma-Aldrich, St. Louis, MO), penicillin (100 IU/mL)/streptomycin (100 μg/mL) (ATCC, Manassas, VA), 10 ng/mL basic fibroblast growth factor (bFGF, Invitrogen-Gibco, Life Technologies), and 10 μM/mL Rho-associated coiled coil-forming protein serine/threonine kinase (ROCK) inhibitor, Y-27632 (Sigma-Aldrich). After the first 24 hours, the medium was changed daily using iPSC medium without ROCK inhibitor. Colony formation was visible within 8–10 days. Cells were passaged every 4–5 days using Accutase (Millipore, Billerica, MA).

### Differentiation of piPSCs

Human piPSCs were differentiated to piPSC-RPE cells using a modified protocol by Singh and colleagues [24] and Meyer and coworkers [25]. Briefly, human piPSC colonies were lifted from MEF feeder layers with accutase (1 mg/mL) and grown as uniform embryoid bodies (EBs) by using AggreWell^™^ plates (STEMCELL Technologies, Vancouver, Canada) for 4 days in EB formation medium (STEMCELL Technologies). At day 5, EB medium was replaced with neural induction medium (NIM) containing DMEM/F12 (1:1), 1% N-2 supplements, MEM non-essential amino acids and 2 μg/mL heparin (Sigma-Aldrich). At day 7, suspended EB aggregates were plated onto laminin-coated culture plates to allow them to reattach to the culture plate, whereupon they were grown for an additional 10 days in NIM. At day 16, neural induction medium was replaced with retinal differentiation medium (RDM) containing DMEM/F12 (3:1), 2% B-27 supplement (without retinoic acid), MEM, non-essential amino acids and penicillin/streptomycin. The cells were maintained as adherent cultures in RDM until the appearance of pigmented piPSC-RPE cells. Large patches of pigmented piPSC-RPE cells were micro-dissected, dissociated with trypsin-ethylenediaminetetraacetic acid (EDTA, 0.05%, Invitrogen-Gibco, Life Technologies) and plated onto laminin-coated transwell inserts (Corning Costar, 3460-Clear, 0.4 mm pores, 12 mm inner diameter, polyester membranes). piPSC-RPE cells were cultured on transwell plates with RDM + 10% fetal bovine serum (FBS) for 2 days and then switched to RDM + 2% FBS until the cells were confluent. Thereafter, piPSC-RPE cells were maintained in RDM to allow them to form compact monolayers and re-pigment within 60–90 days.

### ARPE-19 cell culture

Immortalized human RPE (ARPE-19) cells were obtained from ATCC and propagated in DMEM (Invitrogen-Gibco, Life Technologies) containing 10% fetal bovine serum (FBS), 100 IU/mL penicillin, 100 μg/mL streptomycin, 100 μg/mL gentamicin and 2.5 μg/mL amphotericin B (Invitrogen-Gibco, Life Technologies). The cells were incubated in a humidified atmosphere of 5% CO_2_ and 95% air at 37°C.

### Immunohistochemistry

Cells were fixed with 4% paraformaldehyde at 4°C for 15 minutes, then permeabilized by incubation for 5 minutes in 0.1% Triton X-100 in phosphate-buffered saline (PBS) and incubated with 0.1% bovine serum albumin (BSA) and 1% normal goat serum in PBS for 45 minutes. Slides were incubated with various primary monoclonal or polyclonal antibodies for 2–3 h at room temperature, or overnight at 4°C. Cell cultures were washed and incubated for 1 h at 37°C in the dark with rabbit anti-mouse or goat anti-rabbit IgG antibodies conjugated to either Alexa^™^ 594 (red fluorescence) or Alexa^™^ 488 (green fluorescence) (Invitrogen-Gibco, Life Technologies). Nuclei were stained with diamidinophenyl indole (DAPI) by incubating the slides in the dye solution for 2 minutes. For a summary of immunocytochemistry procedures, see Table 1. The slides were then washed four-times in PBS. Immunologically-stained cell cultures were visualized by fluorescent microscopy (Olympus IX70, Olympus America, Inc., Center Valley, PA) with an attached digital camera (Olympus MicroFire^™^, Olympus America, Inc.) and by a Zeiss 510 NLO confocal laser scanning microscope (Medical University of South Carolina Cell and Molecular Imaging Shared Resource, Carl Zeiss Microscopy GmbH, Jena, Germany) using a Plan-Apochromat 63x/1.4 oil DIC objective.

**Table 1:** List of antibodies used for staining target cells or tissues.

Antibody	Company	Dilution	Target Cells or Tissues
SSEA-4	Millipore-Chemicon, Billerica, MA	1:100	Undifferentiated hESC
TRA-1-60	Millipore-Chemicon, Billerica, MA	1:100	Undifferentiated hESC
Nanog	Millipore-Chemicon, Billerica, MA	1:200	Undifferentiated hESC
ZO-1	Invitrogen-Gibco, Life Technologies, Grand Island, NY	1:100	RPE cell (junction)
Bestrophin	Millipore-Chemicon, Billerica, MA	1:200	RPE cell
MITF	Cell Signaling Technology, Inc., Danvers, MA	1:100	RPE cell
RPE65	Millipore-Chemicon, Billerica, MA	1:200	RPE cell

SSEA-4, stage-specific embryonic antigen-4; TRA-1-60, keratin sulfate-related antidgens-1-60; ZO-1, zonula occludens-1;CRALBP, cellular retinaldehyde-binding protein; MITF, microphthalmia-associated transcription factor; RPE65, retinal pigment epithelium specific protein-65; hESC, human embryonic stem cells; RPE, retinal pigment epithelial cell.

### Isolation of bovine rod outer segments and rhodopsin determination

Bovine rod outer segment (ROS) disk membranes were prepared from frozen retinas as described previously [26]. Outer segment concentration was assessed from the rhodopsin concentration measured spectrophotometrically (Varian Cary 300 Bio UV-Visible) in 0.1% Ammonyx LO (Sigma-Aldrich) [27].

### Fluorescent-labeling of isolated bovine rod outer segments

Isolated ROS disk membranes were labeled with fluorescein isothiocyanate (FITC, Sigma-Aldrich, Cat# F7250) according to manufacturer’s specifications and similar to a previous protocol [28]. Briefly, isolated ROS membranes were pelleted in Hank’s balanced salt solution (HBSS, Invitrogen-Gibco, Life Technologies), and then suspended in 0.1 M sodium bicarbonate buffer, pH 9–9.5. FITC was solubilized in dimethyl sulfoxide (DMSO, Sigma-Aldrich) to a concentration of 2 mg/mL, added to ROS to a final concentration of 50 ng/mL and subsequently incubated for 1 hour at room temperature in the dark with gentle shaking. FITC-stained rod outer segments were washed twice, pelleted in a micro-centrifuge tube (4 min at 4,500 *g*), and re-suspended in growth medium at a concentration of 0.4 μg/μL. These fluorescently-labeled ROS were then seeded to cultured cells in 96-well plates at a rhodopsin concentration of 4–5 μg/cm^2^ or about 1.5 x 10^5^ particles/cm^2^.

### Incubation of piPSC-RPE cells with FITC-labeled rod outer segments

piPSC-RPE cells were seeded onto laminin-coated dishes (Becton Dickinson and Company, BD, Franklin Lakes, NJ) and cultured until confluent. Cells were then overlaid with FITC-labeled ROS, at the concentration mentioned above in growth medium and incubated at 37°C overnight. Cultures were then rinsed with growth medium to remove excess FITC-ROS and subsequently processed for flow cytometry or fixed using 2% paraformaldehyde in 0.1 M phosphate buffer at pH 7.4 for microscopy imaging.

### Flow cytometry

The fluorescence (excitation = 488 nm, emission = 530 nm) of 10,000 unfixed cells/well was assayed immediately on a FACScan (Cyan ADP 9 –color cellular analyzer, Beckman Coulter, Brea, CA) using a live gate to exclude cell fragments, ROS particles, and other unwanted debris. A logarithmic scale of relative fluorescent intensity was used, and ROS phagocytosis was calculated by subtracting the geometric mean autofluorescence of control cells from the geometric mean fluorescence of cells challenged with FITC-labeled ROS. Cellular autofluorescence was determined for each population by analyzing cells from wells with no added ROS. Following ROS challenge, unbound and cell membrane surface-bound ROS were removed by washing the wells three-times with PBS. The bound cells were treated for 10 minutes with 0.25% trypsin containing 1 mM EDTA, pelleted, washed again with PBS, and suspended in 500 μL of PBS with 10 mM glucose at pH 8.0.

### IntegrinαVβ5 antibody-blocking experiments

piPSC-RPE cells were seeded in triplicate on laminin-coated wells in a 96-well plate and allowed to grow to confluence for 3 weeks. Cells were pre-incubated with or without 50 μg/mL anti-integrin αVβ5 (Abcam, Cambridge, United Kingdom) or 50 μg/mL IgG1 control (Abcam, Cambridge, United Kingdom) for 45 minutes, then the medium was replaced and cells were incubated with FITC-labeled ROS (at concentrations as stated above) with or without 50 μg/mL anti-integrin αVβ5, or 50 μg/mL IgG1 control for 2.5 hours at 37°C. Wells were then vigorously washed 3-times with PBS to remove unbound ROS. Fluorescent images were obtained randomly from several fields in the DAPI, FITC wavelength for statistical analysis. Immunocytochemistry of RPE cell marker ZO-1 was performed to visualize cell borders after taking the images.

### Data analysis

The percentage of fluorescently-labelled cells from the total cell frequency count is presented as the mean ± standard error of the mean (SEM). The effect of cell type on phagocytosis was tested using one-way ANOVA. Comparisons between piPSCs, piPSC-RPE cells, ARPE-19 cells and control groups were conducted using the Holm-Sidak post hoc tests, which account for family-wise error. An alpha criterion value of α = 0.05 was adopted.

## Results

### piPSC maintained pluripotency after subcultures were positive for pluripotency markers

**Fig 1:**
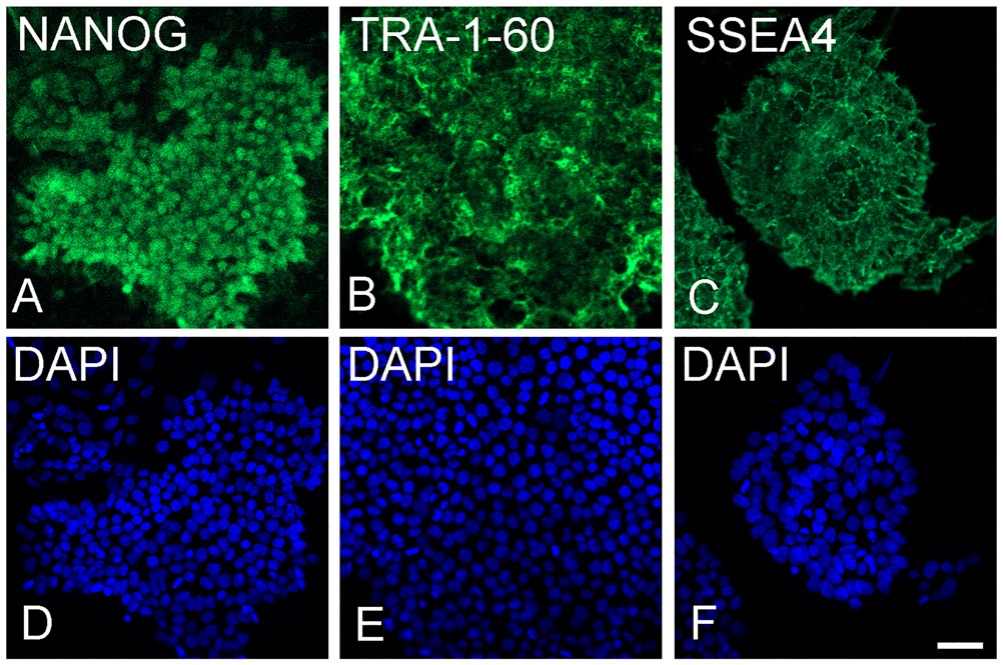
piPSC stained for pluripotency markers after culturing onto mouse embryonic fibroblasts. Immune fluorescence staining was positive for pluripotency markers NANOG (**A**), TRA-1-60 (**B**), SSEA-4 (**C**). Nuclei of **A**-**C** stained with DAPI (**D**-**F**). Bar = 50 μm.

At day 0, immunocytochemistry was used to assess the expression of stem cell markers in the piPSC grown on MEF. These cells were positive for the pluripotent markers NANOG, TRA-1-60 and SSEA-4 (Fig 1).

### Differentiated piPSC-RPE cells expressed RPE cell markers

**Fig 2:**
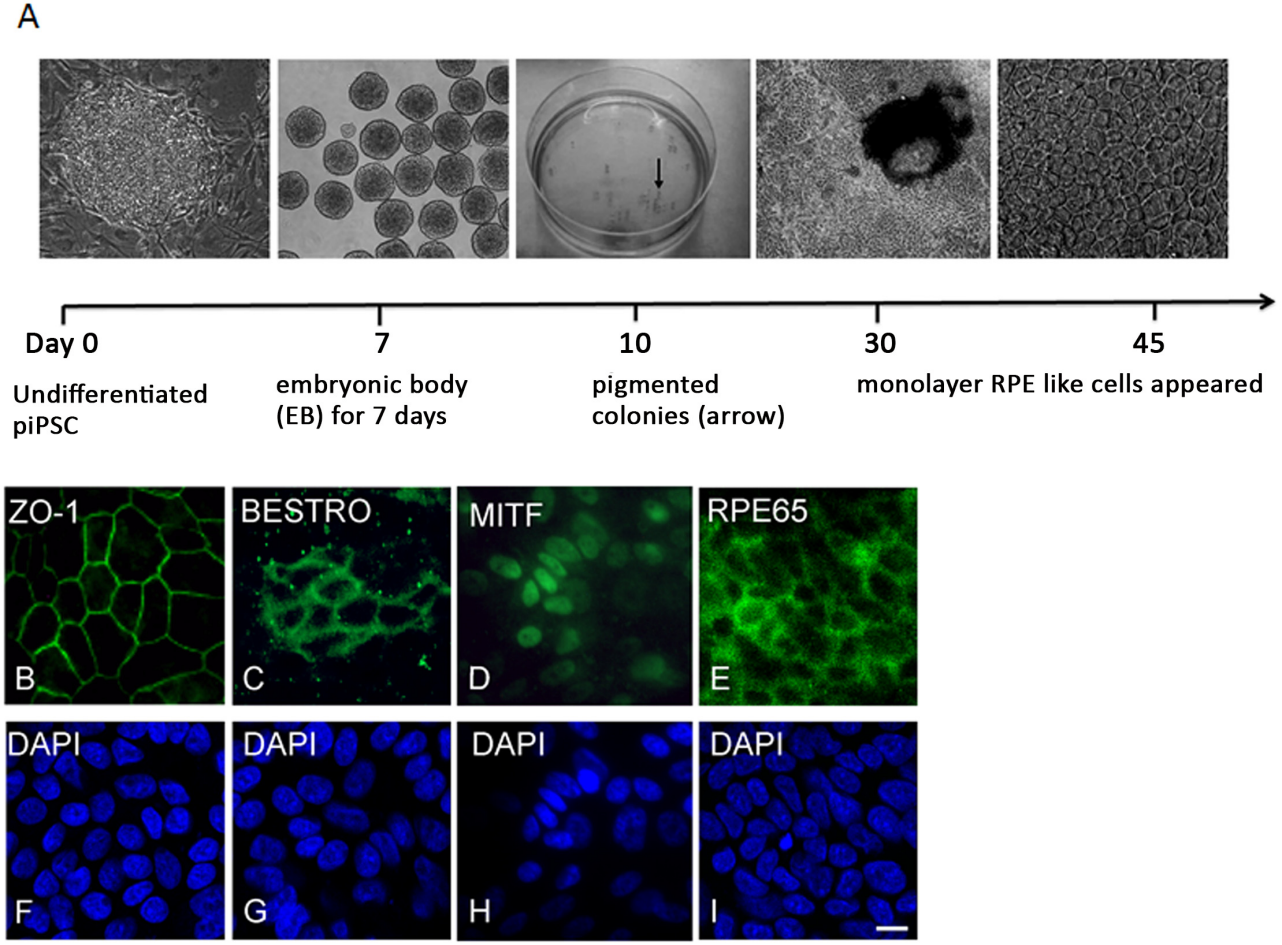
Generation of piPSC-RPE cells. Schematic of the differentiation protocol used to generate piPSC-RPE cells (**A**). Differentiated piPSC-RPE cells were visible after 45 days. Induction of RPE cell markers in piPSC culturing on laminin-coated dishes for 45 days. Immune fluorescence staining was positive for ZO-1 (**B**), bestrophin (**C**), MITF (**D**), RPE65 (**E**). Nuclei of **B**-**E** stained with DAPI (**F-I**). Bar = 10 μm.

After 30 days of differentiation, RPE-like cells were visible, expressing the hexagonal monolayer RPE cell phenotype (Fig 2). After 45 days, differentiated piPSC-RPE cells robustly expressed the tight-junction marker ZO-1, and the RPE cell marker, bestrophin (Fig 2B and 2C). Additionally, the differentiated piPSC-RPE cells expressed MITF and the visual cycle protein, RPE65 (Fig 2D and 2E).

### piPSC-RPE cells perform phagocytic function

**Fig 3:**
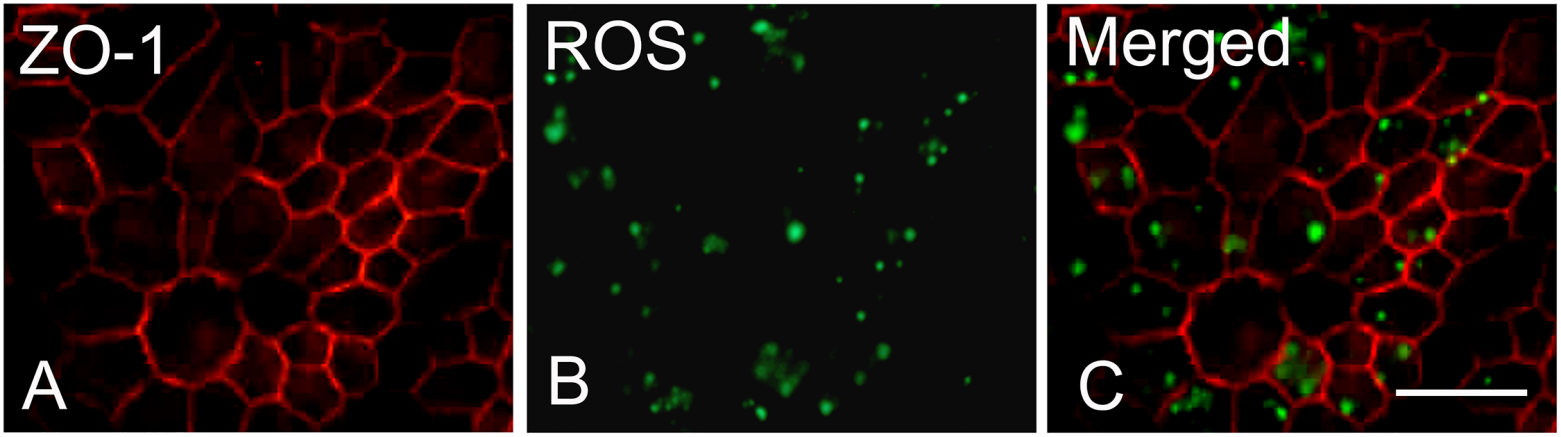
Uptake of FITC-labeled ROS by piPSC-RPE cells after 2.5 hours. (Red): ZO-1 expression on piPSC-RPE cells demonstrating tight junction formation (**A**). (Green): FITC-labeled ROS (**B**). Merged-image demonstrating the phagocytized ROS in RPE cell cytoplasm (**C**). Bar = 50 μm.

Given the expression of RPE cell specific-markers on piPSC-RPE cells, the ability of these cells to phagocytize ROS was explored. We labeled cells with ZO-1, which delineates the cell border to first demonstrate that the phagocytized particles were localized within the cytoplasm (Fig 3A–3C).

**Fig 4:**
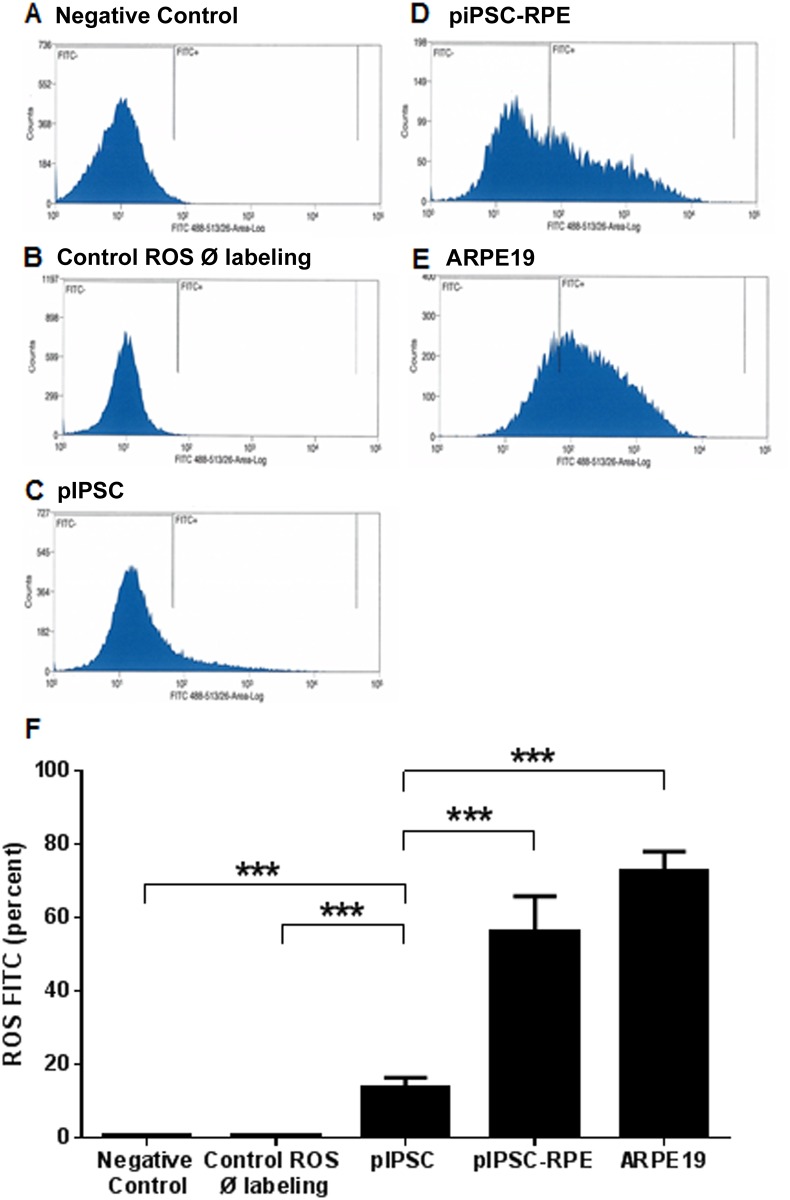
Flow cytometry analysis and percentage of cells containing phagocytized FITC-labeled ROS. Flow cytometry data (**A**) and the histograms (panels **B**-**E**) used to generate that data. Negligible immunofluorescence was detected when either no ROS or only unlabeled ROS were added to piPSC-derived RPE (first 2 bars of **F**). A small amount of immunofluorescence was detected when labelled ROS were added to undifferentiated piPSC. Extensive labelling was noted when labelled ROS were fed to either piPSC-derived RPE or to ARPE-19 (last 2 bars of **F**). There was a significant effect per cell type on phagocytosis F_4,17_ = 34.23, *p* < 0.001 (**F**).

Next we compared the percent of fluorescently-labelled ROS in piPSC, piPSC-RPE cells and ARPE-19 cells after a 16-hour incubation period (Fig 4). There was no significant difference in the amount of fluorescently-labelled ROS between piPSC-RPE cells and ARPE19 cells. Additionally, relative to undifferentiated piPSCs, piPSC-RPE cells had 4.12-times more fluorescently-labelled ROS (iPSC: 13.62 ± 2.74 versus piPSC-RPE: 56.16 ± 9.74; Fig 4).

**Fig 5:**
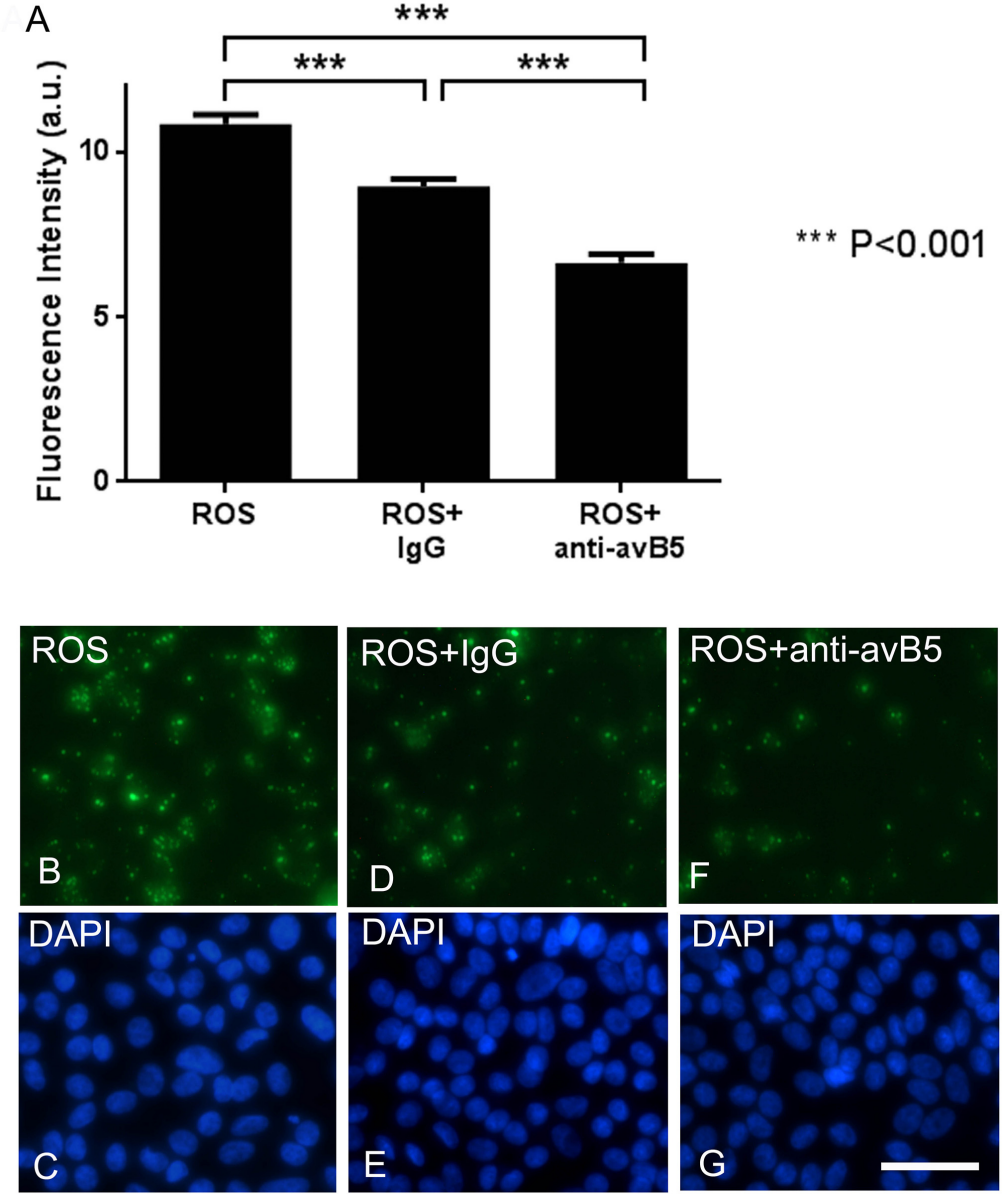
Integrin αVβ5 antibody inhibits the uptake of ROS by piPSC-RPE cells. Baseline uptake of ROS by piPSC-RPE cells was decreased by the addition of a nonspecific monoclonal antibody, IgG1 (**A**). Addition of an inhibitory anti-integrin αVβ5 antibody significantly decreased phagocytosis by approximately 50% in piPSC-RPE cells. Bars represent standard error of the mean. Panels **B**, **D**, **F** show the representative fluorescent images used to generate panel **A**. **C**, **E** and **G** show corresponding DAPI stains for each panel. Abbreviations: anti-avB5, blocking anti-integrin αVβ5 antibody; IgG, isotype-matched immunoglobulin G control antibody; ROS, photoreceptor rod outer segment, and a.u., arbitrary units. Bar = 50 μm.

The αVβ5 integrin receptor is required for photoreceptor rod outer segment uptake [29, 30]. In order to confirm a direct role of the αVβ5 cell surface receptor in the apparent phagocytosis of piPSC-RPE cells, we blocked αVβ5 using a specific antibody applied to ROS. Fluorescently-labeled ROS were incubated with or without anti-integrin αVβ5, or IgG1 control, with piPSC-RPE cells for 2.5 hours. Untreated ROS phagocytosed by piPSC-RPE cells were abundant in the cytoplasm of piPSC (Fig 5B), and its associated fluorescence intensity was 10.84 ± 0.29 arbitrary units (Fig 5A). In contrast, the addition of the blocking antibody, anti-integrin αVβ5, reduced the fluorescence intensity of ROS in piPSC-RPE cells to 6.70 ± 0.25 arbitrary units, such that it was significantly lower than both the untreated piPSC-RPE cell group and ROS control IgG antibody group (*p* < 0.001, Holm Sidak). Addition of the control IgG antibody reduced ROS fluorescence intensity to 8.96 ± 0.22 arbitrary units (*p* < 0.001, Holm Sidak). Therefore, the addition of anti-αVβ5 to ROS significantly reduced the phagocytosis-ability of piPSC-RPE cells relative to untreated piPSC-RPE cells (by 4.14 arbitrary units, *p* < 0.001) and those treated with a control antibody (by 2.26 arbitrary units, *p* < 0.001).

## Discussion

In this study we have confirmed the capacity of piPSCs to differentiate into RPE cells using an RPE cell differentiation protocol established by Meyer and coworkers [25]. We have demonstrated that piPSCs can differentiate toward a RPE cell fate, forming monolayers of piPSC-RPE cells after 30 days in culture. This is comparable to Singh and colleagues [24] who demonstrated the formation of RPE cells from iPSCs after 30–40 days of differentiation, and Buchholz and colleagues [31] who observed pigmented colonies after 20–35 days. We demonstrated the expression of RPE cell markers (ZO-1, bestrophin, MITF, and RPE65) in RPE-like cells differentiated from piPSCs after 45 days in culture. This timing is consistent with previous research which has demonstrated the presence of RPE cell differentiation markers in iPSC-RPE cells after 30 days post differentiation [32]. The presence of RPE65 in piPSCs is of particular importance as it plays a critical role in the retinoid visual cycle and is expressed in terminally differentiated RPE cells [33–35].

To examine whether our piPSC-RPE cells did indeed function as native RPE cells, we used bovine ROS to test the phagocytic activity of piPSC-RPE cells, a characteristic physiological process performed by the RPE cells [36–39]. We demonstrated that piPSC-RPE cells possess the ability to phagocytize bovine ROS at a rate that was over four-times greater compared with undifferentiated piPSC, and was not significantly different to native RPE cells. We observed a small amount of fluorescence-incorporation in undifferentiated piPSC, which may be due to mechanisms independent of the receptor-mediated phagocytosis pathway. Overall, the phagocytic ability of piPSC-RPE cells was very similar to work previously published by Maruotti and coworkers [40] demonstrating apical staining of ZO-1 and phagocytosis of pH-Rhodo-labeled bioparticles in human iPSC-RPE cells. We demonstrated similar results showing ZO-1 staining at the cell margins and intracellular expression of ROS. RPE cells are known to utilize integrin αVβ5 in the initial binding of outer segments prior to internalization [29, 30]. Indeed, our results showed that integrin αVβ5 plays a role in the phagocytic mechanism of these protein-iPSC-derived RPE cells, as we could block these effects by using a specific anti-αVβ5 antibody. Although using a nonspecific IgG antibody had a partial blocking effect, this observation has been reported in other publications, and the mechanism still requires clarification [31]. Taken together, these data suggest that piPSC-RPE cells phagocytize outer segments via an αVβ5-mediated process, at rates comparable to ARPE-19 cells.

Developing new treatments for diseases requiring RPE replacement is of critical importance, but current cell replacement strategies are hindered by allograft rejection, and the requirement for immune suppression. In principle, use of piPSCs may be an attractive alternative for cell replacement therapy despite the low efficiency of protein-based protocols. A priori we would expect that piPSC may be safer than cells generated by introducing foreign DNA into the host cell. However, karyotype or genomic aberrations can also develop during maintenance or passaging of these cells. Although some of these mutations can be irrelevant, silent mutations may affect the cell safety profile [41]. Derivation and subsequent culturing of piPSCs can introduce karyotypic abnormalities in up to 40% of derived cell lines no matter how the cells are derived, although there are differences in the type of alterations introduced using different reprogramming techniques [42]. Therapeutic use of these cells will require further delineation of all genetic and epigenetic alterations introduced, as well as selection of cells with minimal genetic and epigenetic alterations for therapeutic use. Moreover, the timing of RPE cell transplantation and the effect of a non-ideal environment such as aging-host Bruch’s membrane, can potentially affect adherence and long-term survival of transplanted cells [43–45]. Current research is investigating the use of physical supports such as natural and synthetic scaffolds that may improve outcomes of RPE transplantation [44, 46]. Scaffold technology combined with the use of iPSC-derived RPE grafts to slow the progression of disease and prevent photoreceptor cell death may be a viable clinical option for successful treatment of retinal degenerative disorders by enhancing survival and function [45, 47, 48].

## Conclusions

Our results confirm that both morphologically and functionally, piPSCs can be differentiated into RPE cells. Differentiated piPSC-RPE cells expressed typical RPE cell markers and phagocytized ROS via an αVβ5-mediated process. These results provide the initial step toward the development of a new stem cell-derived RPE cell therapy, free from the risk of viral manipulation of the host genome.

## Abbreviations

iPSC: induced pluripotent stem cell
piPSC: protein-induced iPSC
AMD: age-related macular degeneration
RPE: retinal pigment epithelial cell
ESC: embryonic stem cell ESC
ZO-1: zonula occludens protein-1
RPE65: retinal pigment epithelium specific protein-65
MITF: microphthalmia-associated transcription factor
MERTK: c-mer proto-oncogene tyrosine kinase
ROS: rod outer segments
DNA: deoxyribonucleic acid
EBs: embryoid bodies
GA: geographic atrophy

## References

[1] Friedman DS, O'Colmain BJ, Munoz B, Tomany SC, McCarty C, de Jong PT, et al Prevalence of age-related macular degeneration in the United States. Archives of ophthalmology. 2004;122(4):564–72. Epub 2004/04/14. 10.1001/archopht.122.4.564 . 15078675

[2] Nowak JZ. Age-related macular degeneration (AMD): pathogenesis and therapy. Pharmacological reports: PR. 2006;58(3):353–63. Epub 2006/07/18. . 16845209

[3] Jager RD, Mieler WF, Miller JW. Age-related macular degeneration. The New England journal of medicine. 2008;358(24):2606–17. 10.1056/NEJMra0801537 . 18550876

[4] Steinberg RH. The relationship of the retinal pigment epithelium to photoreceptor outer segments in human retina In: Zinn KM, Marmor M.F., editor. The Retinal Pigment Epithelium. Cambridge, MA: Harvard University Press; 1979 p. 32–44.

[5] Hogan MJ. Histology of the Human Eye: WB Saunders Company, Philadelphia; 1971.

[6] Kortvely E, Hauck SM, Duetsch G, Gloeckner CJ, Kremmer E, Alge-Priglinger CS, et al ARMS2 is a constituent of the extracellular matrix providing a link between familial and sporadic age-related macular degenerations. Investigative ophthalmology & visual science. 2010;51(1):79–88. Epub 2009/08/22. 10.1167/iovs.09-3850 . 19696174

[7] Kanda A, Chen W, Othman M, Branham KE, Brooks M, Khanna R, et al A variant of mitochondrial protein LOC387715/ARMS2, not HTRA1, is strongly associated with age-related macular degeneration. Proceedings of the National Academy of Sciences of the United States of America. 2007;104(41):16227–32. Epub 2007/09/22. 10.1073/pnas.0703933104 17884985 PMC1987388

[8] Dewan A, Liu M, Hartman S, Zhang SS, Liu DT, Zhao C, et al HTRA1 promoter polymorphism in wet age-related macular degeneration. Science. 2006;314(5801):989–92. Epub 2006/10/21. 10.1126/science.1133807 . 17053108

[9] Yang Z, Camp NJ, Sun H, Tong Z, Gibbs D, Cameron DJ, et al A variant of the HTRA1 gene increases susceptibility to age-related macular degeneration. Science. 2006;314(5801):992–3. Epub 2006/10/21. 10.1126/science.1133811 . 17053109

[10] Schwartz SD, Regillo CD, Lam BL, Eliott D, Rosenfeld PJ, Gregori NZ, et al Human embryonic stem cell-derived retinal pigment epithelium in patients with age-related macular degeneration and Stargardt's macular dystrophy: follow-up of two open-label phase 1/2 studies. Lancet. 2014 Epub 2014/12/03. 10.1016/S0140-6736(14)61376-3 . 25458728

[11] Schwartz SD, Hubschman J-P, Heilwell G, Franco-Cardenas V, Pan CK, Ostrick RM, et al Embryonic stem cell trials for macular degeneration: a preliminary report. The Lancet. 379(9817):713–20. 10.1016/S0140-6736(12)60028-2 22281388

[12] Algvere PV, Gouras P, Dafgard Kopp E. Long-term outcome of RPE allografts in non-immunosuppressed patients with AMD. European journal of ophthalmology. 1999;9(3):217–30. Epub 1999/11/02. 10544978 10.1177/112067219900900310

[13] Vugler A, Carr AJ, Lawrence J, Chen LL, Burrell K, Wright A, et al Elucidating the phenomenon of HESC-derived RPE: anatomy of cell genesis, expansion and retinal transplantation. Experimental neurology. 2008;214(2):347–61. Epub 2008/10/18. 10.1016/j.expneurol.2008.09.007 . 18926821

[14] Osakada F, Ikeda H, Mandai M, Wataya T, Watanabe K, Yoshimura N, et al Toward the generation of rod and cone photoreceptors from mouse, monkey and human embryonic stem cells. Nature biotechnology. 2008;26(2):215–24. Epub 2008/02/05. 10.1038/nbt1384 . 18246062

[15] Haruta M, Sasai Y, Kawasaki H, Amemiya K, Ooto S, Kitada M, et al In vitro and in vivo characterization of pigment epithelial cells differentiated from primate embryonic stem cells. Investigative ophthalmology & visual science. 2004;45(3):1020–5. Epub 2004/02/27. . 14985325 10.1167/iovs.03-1034

[16] Klimanskaya I, Hipp J, Rezai KA, West M, Atala A, Lanza R. Derivation and comparative assessment of retinal pigment epithelium from human embryonic stem cells using transcriptomics. Cloning and stem cells. 2004;6(3):217–45. Epub 2005/01/27. 10.1089/clo.2004.6.217 . 15671670

[17] Yu J, Vodyanik MA, Smuga-Otto K, Antosiewicz-Bourget J, Frane JL, Tian S, et al Induced Pluripotent Stem Cell Lines Derived from Human Somatic Cells. Science (New York, NY). 2007;318(5858):1917–20. 10.1126/science.1151526 18029452

[18] Takahashi K, Yamanaka S. Induction of pluripotent stem cells from mouse embryonic and adult fibroblast cultures by defined factors. Cell. 2006;126(4):663–76. Epub 2006/08/15. 10.1016/j.cell.2006.07.024 . 16904174

[19] Okita K, Ichisaka T, Yamanaka S. Generation of germline-competent induced pluripotent stem cells. Nature. 2007;448(7151):313–7. Epub 2007/06/08. 10.1038/nature05934 . 17554338

[20] Yamanaka S. Strategies and new developments in the generation of patient-specific pluripotent stem cells. Cell stem cell. 2007;1(1):39–49. Epub 2008/03/29. 10.1016/j.stem.2007.05.012 . 18371333

[21] Kim D, Kim CH, Moon JI, Chung YG, Chang MY, Han BS, et al Generation of human induced pluripotent stem cells by direct delivery of reprogramming proteins. Cell stem cell. 2009;4(6):472–6. Epub 2009/06/02. 10.1016/j.stem.2009.05.005 19481515 PMC2705327

[22] Rhee YH, Ko JY, Chang MY, Yi SH, Kim D, Kim CH, et al Protein-based human iPS cells efficiently generate functional dopamine neurons and can treat a rat model of Parkinson disease. The Journal of clinical investigation. 2011;121(6):2326–35. Epub 2011/05/18. 10.1172/JCI45794 21576821 PMC3104759

[23] Gonzalez F, Boue S, Izpisua Belmonte JC. Methods for making induced pluripotent stem cells: reprogramming a la carte. Nature reviews Genetics. 2011;12(4):231–42. Epub 2011/02/23. 10.1038/nrg2937 . 21339765

[24] Singh R, Shen W, Kuai D, Martin JM, Guo X, Smith MA, et al iPS cell modeling of Best disease: insights into the pathophysiology of an inherited macular degeneration. Human molecular genetics. 2013;22(3):593–607. Epub 2012/11/10. 10.1093/hmg/dds469 23139242 PMC3542866

[25] Meyer JS, Shearer RL, Capowski EE, Wright LS, Wallace KA, McMillan EL, et al Modeling early retinal development with human embryonic and induced pluripotent stem cells. Proceedings of the National Academy of Sciences of the United States of America. 2009;106(39):16698–703. Epub 2009/08/27. 10.1073/pnas.0905245106 19706890 PMC2757802

[26] Papermaster DS, Dreyer WJ. Rhodopsin content in the outer segment membranes of bovine and frog retinal rods. Biochemistry. 1974;13(11):2438–44. Epub 1974/05/21. . 4545509 10.1021/bi00708a031

[27] Fong SL, Tsin AT, Bridges CD, Liou GI. Detergents for extraction of visual pigments: types, solubilization, and stability. Methods in enzymology. 1982;81:133–40. Epub 1982/01/01. . 6980357 10.1016/s0076-6879(82)81022-7

[28] Sun K, Cai H, Tezel TH, Paik D, Gaillard ER, Del Priore LV. Bruch's membrane aging decreases phagocytosis of outer segments by retinal pigment epithelium. Molecular vision. 2007;13:2310–9. Epub 2008/01/18. . 18199972

[29] Lin H, Clegg DO. Integrin alphavbeta5 participates in the binding of photoreceptor rod outer segments during phagocytosis by cultured human retinal pigment epithelium. Investigative ophthalmology & visual science. 1998;39(9):1703–12. Epub 1998/08/12. . 9699560

[30] Finnemann SC, Bonilha VL, Marmorstein AD, Rodriguez-Boulan E. Phagocytosis of rod outer segments by retinal pigment epithelial cells requires alpha(v)beta5 integrin for binding but not for internalization. Proceedings of the National Academy of Sciences of the United States of America. 1997;94(24):12932–7. Epub 1997/11/25. 9371778 10.1073/pnas.94.24.12932 PMC24241

[31] Buchholz DE, Hikita ST, Rowland TJ, Friedrich AM, Hinman CR, Johnson LV, et al Derivation of functional retinal pigmented epithelium from induced pluripotent stem cells. Stem Cells. 2009;27(10):2427–34. Epub 2009/08/07. 10.1002/stem.189 . 19658190

[32] Singh R, Phillips MJ, Kuai D, Meyer J, Martin JM, Smith MA, et al Functional analysis of serially expanded human iPS cell-derived RPE cultures. Investigative ophthalmology & visual science. 2013;54(10):6767–78. Epub 2013/09/14. 10.1167/iovs.13-11943 24030465 PMC3799561

[33] Moiseyev G, Chen Y, Takahashi Y, Wu BX, Ma JX. RPE65 is the isomerohydrolase in the retinoid visual cycle. Proc Natl Acad Sci U S A. 2005;102(35):12413–8. 10.1073/pnas.0503460102 16116091 PMC1194921

[34] Moiseyev G, Takahashi Y, Chen Y, Gentleman S, Redmond TM, Crouch RK, et al RPE65 is an iron(II)-dependent isomerohydrolase in the retinoid visual cycle. The Journal of biological chemistry. 2006;281(5):2835–40. 10.1074/jbc.M508903200 . 16319067

[35] Thompson DA, Gal A. Vitamin A metabolism in the retinal pigment epithelium: genes, mutations, and diseases. Prog Retin Eye Res. 2003;22(5):683–703. . 12892646 10.1016/s1350-9462(03)00051-x

[36] Mazzoni F, Safa H, Finnemann SC. Understanding photoreceptor outer segment phagocytosis: Use and utility of RPE cells in culture. Experimental eye research. 2014 Epub 2014/05/02. 10.1016/j.exer.2014.01.010 . 24780752 PMC4145030

[37] LaVail MM. Rod outer segment disk shedding in rat retina: relationship to cyclic lighting. Science. 1976;194(4269):1071–4. Epub 1976/12/03. . 982063 10.1126/science.982063

[38] Young RW. The daily rhythm of shedding and degradation of cone outer segment membranes in the lizard retina. Journal of ultrastructure research. 1977;61(2):172–85. Epub 1977/11/01. . 562420 10.1016/s0022-5320(77)80084-1

[39] Young RW, Bok D. Participation of the retinal pigment epithelium in the rod outer segment renewal process. The Journal of cell biology. 1969;42(2):392–403. Epub 1969/08/01. 5792328 10.1083/jcb.42.2.392 PMC2107669

[40] Maruotti J, Wahlin K, Gorrell D, Bhutto I, Lutty G, Zack DJ. A simple and scalable process for the differentiation of retinal pigment epithelium from human pluripotent stem cells. Stem Cells Transl Med. 2013;2(5):341–54. 10.5966/sctm.2012-0106 23585288 PMC3667560

[41] Lund RJ, Narva E, Lahesmaa R. Genetic and epigenetic stability of human pluripotent stem cells. Nature reviews Genetics. 2012;13(10):732–44. 10.1038/nrg3271 . 22965355

[42] Martins-Taylor K, Xu RH. Concise review: Genomic stability of human induced pluripotent stem cells. Stem Cells. 2012;30(1):22–7. 10.1002/stem.705 . 21823210

[43] Heller JP, Martin KR. Enhancing RPE Cell-Based Therapy Outcomes for AMD: The Role of Bruch's Membrane. Translational vision science & technology. 2014;3(3):11 10.1167/tvst.3.3.11 25068093 PMC4108298

[44] Tezel TH, Kaplan HJ, Del Priore LV. Fate of human retinal pigment epithelial cells seeded onto layers of human Bruch's membrane. Investigative ophthalmology & visual science. 1999;40(2):467–76. Epub 1999/02/09. . 9950607

[45] Sorkio A, Porter PJ, Juuti-Uusitalo K, Meenan BJ, Skottman H, Burke GA. Surface Modified Biodegradable Electrospun Membranes as a Carrier for Human Embryonic Stem Cell-Derived Retinal Pigment Epithelial Cells. Tissue engineering Part A. 2015;21(17–18):2301–14. 10.1089/ten.TEA.2014.0640 . 25946229

[46] Sugino IK, Sun Q, Wang J, Nunes CF, Cheewatrakoolpong N, Rapista A, et al Comparison of FRPE and human embryonic stem cell-derived RPE behavior on aged human Bruch's membrane. Investigative ophthalmology & visual science. 2011;52(8):4979–97. 10.1167/iovs.10-5386 21460262 PMC3176062

[47] Diniz B, Thomas P, Thomas B, Ribeiro R, Hu Y, Brant R, et al Subretinal implantation of retinal pigment epithelial cells derived from human embryonic stem cells: improved survival when implanted as a monolayer. Investigative ophthalmology & visual science. 2013;54(7):5087–96. 10.1167/iovs.12-11239 23833067 PMC3726243

[48] Treharne AJ, Thomson HA, Grossel MC, Lotery AJ. Developing methacrylate-based copolymers as an artificial Bruch's membrane substitute. Journal of biomedical materials research Part A. 2012;100(9):2358–64. 10.1002/jbm.a.34178 . 22528296

